# On Almost Norden Statistical Manifolds

**DOI:** 10.3390/e24060758

**Published:** 2022-05-27

**Authors:** Leila Samereh, Esmaeil Peyghan, Ion Mihai

**Affiliations:** 1 Department of Mathematics, Faculty of Science, Arak University, Arak 38156-8-8349, Iran; l.samereh@yahoo.com (L.S.); e-peyghan@araku.ac.ir (E.P.); 2 Department of Mathematics, Faculty of Mathematics and Computer Science, University of Bucharest, 010014 Bucharest, Romania

**Keywords:** almost Norden manifold, dual connections, statistical manifold, complex conjugate connections, almost complex structure, 53C05, 53C15

## Abstract

We consider a statistical connection ∇ on an almost complex manifold with (pseudo-) Riemannian metric, in particular the Norden metric. We investigate almost Norden (statistical) manifolds under the condition that the almost complex structure *J* is ∇-recurrent. We provide one example of a complex statistical connection.

## 1. Introduction

Recently, the study of spaces consisting of probability measures is receiving more attention. Information geometry, as a famous theory in geometry, is a tool to investigate such spaces (of course in the finite-dimensional sense). Information geometry as a combination of differential geometry and statistics has an important role in science. For example, image processing, physics, computer science, and machine learning are some of its applications (see [1,2,3,4]). From one point of view, it is a realm that makes it possible to illustrate statistical objects as geometric ones by the way of capturing their geometric properties.

For an open subset Θ of $R^{n}$ and a sample space Ω with parameter $\theta =(\theta^{1},\cdots ,\theta^{n})$, we call the set of probability density functions

$$S=\{p\left(x;\theta \right):\int_{\Omega }p\left(x;\theta \right)=1,\;\;p\left(x;\theta \right)>0,\;\;\theta \in \Theta \subseteq R^{n}\},$$

as a statistical model. For a statistical model *S*, the semi-definite Fisher information matrix $g\left(\theta \right)=[g_{ij}\left(\theta \right)]$ is defined as

(1) $$g_{ij}\left(\theta \right):=\int_{\Omega }\partial_{i}\ell_{\theta }\partial_{j}\ell_{\theta }p\left(x;\theta \right)dx=E_{p}\left[\partial_{i}\ell_{\theta }\partial_{j}\ell_{\theta }\right],$$

where $\ell_{\theta }=\ell \left(x;\theta \right):=\log p\left(x;\theta \right)$, $\partial_{i}:=\frac{\partial }{\partial \theta^{i}}$, and $E_{p}\left[f\right]$ is the expectation of *f* with respect to p(x;θ). Equipping the space *S* with such information matrixes, it becomes a statistical manifold.

Fisher was the first to introduce the relation (1) as a mathematical intent of information in 1920 (see [5]). It is shown that if *g* is positive-definite and all of its components are converging to real numbers, then (S,g) will be a Riemannian manifold and *g* is called a Fisher metric on *S* with components

$$\begin{array}{cc} g_{ij}\left(\theta \right) & =\int_{\Omega }\partial_{i}p\left(x;\theta \right)\partial_{j}\ell_{\theta }dx=\int_{\Omega }\frac{1}{p(x;\theta )}\partial_{i}p\left(x;\theta \right)\partial_{j}p\left(x;\theta \right)dx. \end{array}$$

Rao was the first to study the above metric in 1945 (see [6]).

By a statistical manifold we mean a triple (M,g,∇), where the manifold *M* is equipped with a statistical structure (g,∇) containing a (pseudo) Riemannian metric *g* and a linear connection ∇ on *M* such that the covariant derivative ∇g is symmetric.

Recently, statistical manifolds have attracted the attention of many mathematicians (see for instance [7,8,9,10]). A fundamental role in characterizing statistical manifolds is played by two geometric quantities, called dual connections, which describe the derivation with respect to vector fields and are interrelated in a duality relation involving the Fisher metric. The study of dual elements and the relations between them constitute the main direction of development in the study of statistical manifolds [11].

Hermitian manifolds as well as Norden manifolds have been studied from various points of view. Here we refer to [9,12,13,14]. Since on Norden manifolds, there exists a pair of Norden metrics, one can consider dual (conjugate) connections with respect to each of these metrical tensors and their relations to dual connections relative to the almost complex structure [14]. Therefore, the study of statistical structures on these manifolds is of great importance.

The main purpose of this paper is to study almost Norden manifolds with statistical connections. The paper is organized as follows. In Section 2, we recall some basic concepts about statistical geometry. In Section 3, the main focus is on almost Norden manifolds with statistical connections. Furthermore, we obtain some results about the skewness tensor *K*. In Section 4, we concentrate mainly on almost complex structures *J* which are ∇-recurrent. This condition lets us study some kinds of almost complex statistical manifolds. In the last section, we construct one complex statistical connection.

## 2. Preliminaries

Let *M* be a smooth manifold with a (pseudo) Riemannian metric *g* and ∇ be a symmetric linear connection.

The triple (M,g,∇) is called a *statistical manifold* [15] if ∇g is symmetric, i.e., ∇g=C, where *C* is a symmetric tensor of degree (0,3), namely ∇g satisfies the Codazzi equation

(2) $$\begin{array}{c} \left(\nabla_{X}g\right)\left(Y,Z\right)=\left(\nabla_{Y}g\right)\left(X,Z\right)=\left(\nabla_{Z}g\right)\left(Y,X\right)=C\left(X,Y,Z\right),\forall X,Y,Z\in \Gamma \left(TM\right). \end{array}$$

In this case, ∇ is called a statistical connection and the pairing (∇,g) a statistical structure on *M* (see [15]). When C=0, we have the unique Levi-Civita connection $\nabla^{\left(0\right)}$. The dual connection $\nabla^{*}$ of a linear connection ∇ is defined by

(3) $$\begin{array}{c} Xg\left(Y,Z\right)=g\left(\nabla_{X}Y,Z\right)+g\left(Y,\nabla_{X}^{*}Z\right),\;\;\;\;\forall X,Y,Z\in \Gamma \left(TM\right). \end{array}$$

Now we define the *skewness operator* *K* of degree (1,2) on *M* as follows:

(4) $$\begin{array}{c} K\left(X,Y\right)=\nabla_{X}Y-\nabla_{X}^{*}Y,\;\;\;\;\;\forall X,Y\in \Gamma \left(TM\right). \end{array}$$

It is easy to see that *K* satisfies the following.

(i)
K(X,Y)=K(Y,X),

(ii)
g(K(X,Y),Z)=g(Y,K(X,Z)),

(iii) C(X,Y,Z)=−g(K(X,Y),Z),∀X,Y,Z∈Γ(TM).

It is known that if (M,g,∇) is a statistical manifold, then $\left(M,g,\nabla^{*}\right)$ is a statistical manifold as well [15]. Moreover, we have

(5) $$\begin{array}{c} \nabla^{\left(0\right)}=\frac{1}{2}\left(\nabla +\nabla^{*}\right). \end{array}$$

It is remarkable, from (4) and (5) we have

$$\begin{array}{c} \nabla_{X}Y=\nabla_{X}^{\left(0\right)}Y+\frac{1}{2}K\left(X,Y\right). \end{array}$$

In affine differential geometry, the dual connections are called *conjugate connections* (see [16,17]).

## 3. Statistical Connections on Almost Norden Manifolds

Fei and Zhang proved an important result ([9], Theorem 2.13) about Klein transformation group of conjugate connections.

We will give some related properties of almost Norden statistical manifolds.

Let *M* be a 2n-dimensional differentiable manifold, *J* an almost complex structure and *g* a pseudo-Riemannian metric compatible with *J*, i.e.,

(6) $$\begin{array}{c} J^{2}X=-X,\;\;\;\;\;\;g\left(JX,JY\right)=-g\left(X,Y\right). \end{array}$$

The couple (M,J) is said to be an almost complex manifold and the triple (M,J,g) is called an almost Norden manifold. The triple (M,J,g) is also called an almost complex manifold with Norden metric.

From Equation (6) one obtains g(JX,Y)=g(X,JY); it follows that the tensor $\tilde{g}$ defined by

(7) $$\begin{array}{c} \tilde{g}\left(X,Y\right)=g\left(X,JY\right), \end{array}$$

is symmetric. The tensor $\tilde{g}$ is known as the associated (twin) metric of *g*. It is also a Norden metric, namely it satisfies

$$\begin{array}{c} \tilde{g}\left(JX,JY\right)=-\tilde{g}\left(X,Y\right). \end{array}$$

It is worth noting that the pseudo-Riemannian metrics *g* and $\tilde{g}$ are necessarily of neutral signature (n,n).

**Proposition** **1.** *Ref. [18] Let (M,J,g) be an almost Norden manifold and* ∇ *the Levi-Civita connection of g. Then*

(8) $$\begin{array}{c} g\left(\left(\nabla_{X}J\right)Z,Y\right)=g\left(\left(\nabla_{X}J\right)Y,Z\right). \end{array}$$

An almost Norden manifold (M,J,g) with a statistical connection ∇ is called an almost Norden statistical manifold.

**Proposition** **2.** *Let (M,J,g) be an almost Norden manifold and* ∇ *the Levi-Civita connection of g. If $\left(\nabla ,\tilde{g}\right)$ is a statistical structure, then*

(9) $$\begin{array}{c} \tilde{g}\left(J\left(\nabla_{X}J\right)Z,Y\right)=\tilde{g}\left(J\left(\nabla_{Y}J\right)Z,X\right). \end{array}$$

**Proof.**  For any X,Y,Z∈Γ(TM), we have

$$\begin{array}{cc} \left(\nabla_{X}\tilde{g}\right)\left(Y,Z\right) & =X\tilde{g}\left(Y,Z\right)-\tilde{g}\left(\nabla_{X}Y,Z\right)-\tilde{g}\left(Y,\nabla_{X}Z\right)\\ & =Xg\left(Y,JZ\right)-g\left(\nabla_{X}Y,JZ\right)-g\left(Y,J\left(\nabla_{X}Z\right)\right)\\ & =g\left(\nabla_{X}JZ,Y\right)-g\left(Y,J\left(\nabla_{X}Z\right)\right)\\ & =g\left(\left(\nabla_{X}J\right)Z,Y\right)=-\tilde{g}\left(J\left(\nabla_{X}J\right)Z,Y\right). \end{array}$$

In the same way

$$\begin{array}{c} \left(\nabla_{Y}\tilde{g}\right)\left(X,Z\right)=-\tilde{g}\left(J\left(\nabla_{Y}J\right)Z,X\right). \end{array}$$

Based on the assumption that $\left(M,\tilde{g},\nabla \right)$ is a statistical manifold, we obtain the equality.   □

Now, we consider an almost Norden statistical manifold $\left(M,J,\tilde{g},\nabla \right)$ such that ∇ is the Levi-Civita connection of *g*. Due to the relations (8) and (9), we can define (0,3)-symmetric tensor

$$\begin{array}{c} C\left(X,Y,Z\right)=-\tilde{g}\left(J\left(\nabla_{X}J\right)Y,Z\right). \end{array}$$

Based on the assumption that $\left(M,\tilde{g},\nabla \right)$ is a statistical manifold, it follows that $\nabla_{X}Y={\tilde{\nabla }}_{X}^{\left(0\right)}Y+\frac{1}{2}K\left(X,Y\right)$, where ${\tilde{\nabla }}^{\left(0\right)}$ is the Levi-Civita connection with respect to $\tilde{g}$. By the relation $\left(\nabla_{X}\tilde{g}\right)\left(Y,Z\right)=\left(\nabla_{X}g\right)\left(JY,Z\right)+g\left(\left(\nabla_{X}J\right)Y,Z\right)$ and the above assumption that ∇ is the Levi-Civita connection of *g*, we have $\left(\nabla_{X}J\right)Y=\left(\nabla_{Y}J\right)X$. Therefore we can state the following theorem.

**Theorem** **1.** *Let $\left(M,J,\tilde{g},\nabla \right)$ be an almost Norden statistical manifold and* ∇ *the Levi-Civita connection of g. If the (1,2)-symmetric tensor $K\left(X,Y\right)=J\left(\nabla_{X}J\right)Y$, then*

$$\begin{array}{c} \nabla_{X}Y-{\tilde{\nabla }}_{X}^{\left(0\right)}Y=\frac{1}{2}J\left(\left(\nabla_{X}J\right)Y\right). \end{array}$$

In the following section, we study some properties of the operator *K* on almost Norden statistical manifolds.

**Proposition** **3.**  *Let* (M,J,g) *be an almost Norden statistical manifold. Then*

*(i)* 
$\left(\nabla_{X}J\right)Y-\left(\nabla_{X}^{*}J\right)Y=K\left(X,JY\right)-J\left(K\left(X,Y\right)\right),\forall X,Y\in \Gamma \left(TM\right).$

*(ii)*  If $\left(\nabla_{X}J\right)Y=\left(\nabla_{Y}J\right)X$, then $\left(\nabla_{X}^{*}J\right)Y=\left(\nabla_{Y}^{*}J\right)X$ if and only if K(X,JY)=K(Y,JX),∀X,Y∈Γ(TM).

Indeed,

$$\begin{array}{cc} \left(\nabla_{X}J\right)Y-\left(\nabla_{X}^{*}J\right)Y & =\nabla_{X}JY-J\left(\nabla_{X}Y\right)-\nabla_{X}^{*}JY+J\left(\nabla_{X}^{*}Y\right)\\ & =K(X,JY)-J(K(X,Y)). \end{array}$$

**Proposition** **4.**  *Let $\left(M,J,\tilde{g},\nabla \right)$ be an almost Norden statistical manifold and let* ∇ *be the Levi-Civita connection of g, then*

*(i)*  $\;\left(\nabla_{X}J\right)Y=-J\left(K\left(X,Y\right)\right),\forall X,Y\in \Gamma \left(TM\right)$
*(ii)*  $\;\left(\nabla_{X}^{*}J\right)Y=K\left(X,JY\right),\forall X,Y\in \Gamma \left(TM\right).$

**Proof.** 

(i) Because $\left(\nabla ,\tilde{g}\right)$ is a statistical structure, one has
  $$\begin{array}{c} X\tilde{g}\left(Y,Z\right)=\tilde{g}\left(\nabla_{X}Y,Z\right)+\tilde{g}\left(Y,\nabla_{X}^{*}Z\right), \end{array}$$
  $$\begin{array}{c} Xg\left(Y,JZ\right)=g\left(J\nabla_{X}Y,JZ\right)+g\left(Y,J\left(\nabla_{X}^{*}Z\right)\right). \end{array}$$
  Since ∇ is the Levi-Civita connection of *g*, it follows that
  $$\begin{array}{c} g\left(J\nabla_{X}JZ,Y\right)=g\left(Y,J\left(\nabla_{X}^{*}Z\right)\right), \end{array}$$
  so
  $$\begin{array}{c} \left(\nabla_{X}J\right)Y=\nabla_{X}JY-J\left(\nabla_{X}Y\right)=J\left(\nabla_{X}^{*}Y\right)-J\left(\nabla_{X}Y\right)=-J\left(K\left(X,Y\right)\right). \end{array}$$
(ii) Since
  $$\begin{array}{c} \left(\nabla_{X}J\right)Y-\left(\nabla_{X}^{*}J\right)Y=K\left(X,JY\right)-J\left(K\left(X,Y\right)\right), \end{array}$$
  we obtain $\left(\nabla_{X}^{*}J\right)Y=K\left(X,JY\right).$

□

**Proposition** **5.**  *Let (M,J,g,∇) be an almost Norden statistical manifold and* ∇ *the Levi-Civita connection of $\tilde{g}$. Then*

*(i)*  $\left(\nabla_{X}J\right)Y=K\left(X,JY\right),\forall X,Y\in \Gamma \left(TM\right).$
*(ii)*  $\left(\nabla_{X}^{*}J\right)Y=-J\left(K\left(X,Y\right)\right),\forall X,Y\in \Gamma \left(TM\right).$

**Proof.**  (i) Let X,Y∈Γ(TM). We have

$$K\left(X,JY\right)=J\left(\left(\nabla_{X}J\right)JY\right)=-J\nabla_{X}Y+\nabla_{X}JY=\left(\nabla_{X}J\right)Y.$$

Similarly for statement (ii).   □

**Corollary** **1.** *Let (M,J,g,∇) be an almost Norden statistical manifold satisfying ∇J=0. Then K(JX,Y)=K(JY,X),∀X,Y∈Γ(TM).*

**Corollary** **2.** *Let $\left(M,J,\tilde{g},\nabla \right)$ be an almost Norden statistical manifold and let ∇J=0 then K(JX,Y)=K(JY,X),∀X,Y∈Γ(TM).*

## 4. ∇-Recurrent Almost Complex Structures *J*

In this section, we focus on connections ∇ satisfying, for any X,Y∈Γ(TM), $\left(\nabla_{X}J\right)Y=\tau \left(X\right)JY$, for some 1-form τ, namely the almost complex structure *J* is ∇-recurrent. The notion ∇-recurrent with respect to the almost complex structure *J* was used in [19]. Considering this condition for the linear connection ∇, we study several kinds of almost complex manifolds.

**Proposition** **6.** *Let (M,J,g,∇) be an almost Norden statistical manifold with* ∇*-recurrent almost complex structures J. Then K(JX,Y)=K(JY,X),∀X,Y∈Γ(TM).*

**Proof.**  Since (M,g,∇) is a statistical manifold

$$\begin{array}{c} \left(\nabla_{X}g\right)\left(Y,JZ\right)=\left(\nabla_{Y}g\right)\left(X,JZ\right),\;\forall X,Y,Z\in \Gamma \left(TM\right). \end{array}$$

We have

$$\begin{array}{cc} \left(\nabla_{X}g\right)\left(Y,JZ\right) & =Xg\left(Y,JZ\right)-g\left(\nabla_{X}Y,JZ\right)-g\left(Y,\nabla_{X}JZ\right)\\ & =Xg\left(JY,Z\right)-g\left(\nabla_{X}Y,JZ\right)-g\left(Y,\left(\nabla_{X}J\right)Z\right)-g\left(Y,J\left(\nabla_{X}Z\right)\right)\\ & =g\left(\nabla_{X}^{*}JY,Z\right)-g\left(\nabla_{X}Y,JZ\right)-g\left(Y,\left(\nabla_{X}J\right)Z\right). \end{array}$$

Because $\left(\nabla_{X}J\right)Y=\tau \left(X\right)JY$, we obtain

$$\begin{array}{c} g\left(\left(\nabla_{X}J\right)Y,Z\right)=g\left(\tau \left(X\right)JY,Z\right)=g\left(\tau \left(X\right)JZ,Y\right)=g\left(\left(\nabla_{X}J\right)Z,Y\right). \end{array}$$

Thus

(10) $$\begin{array}{c} \left(\nabla_{X}g\right)\left(Y,JZ\right)=g\left(\nabla_{X}^{*}JY,Z\right)-g\left(J\left(\nabla_{X}Y\right),Z\right)-g\left(Z,\left(\nabla_{X}J\right)Y\right). \end{array}$$

In the same way

(11) $$\begin{array}{c} \left(\nabla_{Y}g\right)\left(X,JZ\right)=g\left(\nabla_{Y}^{*}JX,Z\right)-g\left(J\left(\nabla_{Y}X\right),Z\right)-g\left(Z,\left(\nabla_{Y}J\right)X\right). \end{array}$$

Subtracting (11) from (10), we have

$$\begin{array}{cc} \; & \left(\nabla_{X}g\right)\left(Y,JZ\right)-\left(\nabla_{Y}g\right)\left(X,JZ\right)\\ & =g\left(\nabla_{X}^{*}JY,Z\right)-g\left(J\left[X,Y\right],Z\right)-g\left(\left(\nabla_{X}J\right)Y,Z\right)\\ & -g\left(\nabla_{Y}^{*}JX,Z\right)+g\left(\left(\nabla_{Y}J\right)X,Z\right)=0. \end{array}$$

Since *g* is non-degenerate, it follows that

$$\begin{array}{c} \nabla_{X}^{*}JY-\nabla_{Y}^{*}JX-J\left[X,Y\right]-\left(\nabla_{X}J\right)Y+\left(\nabla_{Y}J\right)X=0; \end{array}$$

then

$$\begin{array}{c} \left(\nabla_{X}^{*}J\right)Y+J\left(\nabla_{X}^{*}Y\right)-\left(\nabla_{Y}^{*}J\right)X-J\left(\nabla_{Y}^{*}X\right)-J\left[X,Y\right]-\left(\nabla_{X}J\right)Y+\left(\nabla_{Y}J\right)X=0. \end{array}$$

Because $\nabla^{*}$ is symmetric,

$$\begin{array}{c} \left(\nabla_{X}^{*}J\right)Y-\left(\nabla_{X}J\right)Y-\left(\nabla_{Y}^{*}J\right)X+\left(\nabla_{Y}J\right)X=0. \end{array}$$

Finally from Proposition 3

$$\begin{array}{c} J(K(X,Y))-K(X,JY)-J(K(Y,X))+K(Y,JX)=0, \end{array}$$

$$\begin{array}{c} K(JX,Y)=K(JY,X). \end{array}$$

□

We will consider an extension of the notion of a statistical structure. Let *M* be a smooth manifold. Consider a tensor *h* of type (0,2) and a linear connection ∇. The triple (M,h,∇) is called a *quasi statistical manifold* if $d^{\nabla }h=0$ [20], where $d^{\nabla }h$ is defined by

(12) $$\begin{array}{c} \left(d^{\nabla }h\right)\left(X,Y,Z\right):=\left(\nabla_{X}h\right)\left(Y,Z\right)-\left(\nabla_{Y}h\right)\left(X,Z\right)+h\left(T^{\nabla }\left(X,Y\right),Z\right). \end{array}$$

If *h* is a pseudo-Riemannian metric, we call (M,h,∇) a statistical manifold admitting torsion [21].

A semi-symmetric linear connection on a differentiable manifold was introduced by Friedmann and Schouten [22]. Let (M,g) be a (pseudo) Riemannian manifold. A linear connection ∇ on *M* is said to be semi-symmetric if its torsion $T^{\nabla }$ is given by

$$\begin{array}{c} T^{\nabla }\left(X,Y\right)=\pi \left(Y\right)X-\pi \left(X\right)Y, \end{array}$$

for X,Y∈Γ(TM). π is a 1-form associated with the vector field *P*, i.e., π(X)=g(X,P). Tao and Zhang in some parts of [23] have studied linear connections with respect to an arbitrary invertible operator on space TM as well as an arbitrary 1-form.

**Proposition** **7.** *Let $\left(M,J,\tilde{g}\right)$ be an almost Norden manifold. Consider a metrical structure (g,∇) such that J is* ∇*-recurrent. Then $\left(M,\tilde{g},\nabla \right)$ is a statistical manifold admitting torsion if and only if it is semi symmetric.*

**Proof.**  Using the relation (12), ∇g=0 and $\left(\nabla_{X}J\right)Y=\tau \left(X\right)JY$, we have

$$\begin{array}{cc} \; & \left(\nabla_{X}\tilde{g}\right)\left(Y,Z\right)-\left(\nabla_{Y}\tilde{g}\right)\left(X,Z\right)\\ & =Xg\left(Y,JZ\right)-g\left(\nabla_{X}Y,JZ\right)-g\left(JY,\nabla_{X}Z\right)\\ & -Yg\left(X,JZ\right)+g\left(\nabla_{Y}X,JZ\right)+g\left(JX,\nabla_{Y}Z\right)\\ & =g\left(Y,\nabla_{X}JZ\right)-g\left(JY,\nabla_{X}Z\right)-g\left(X,\nabla_{Y}JZ\right)+g\left(JX,\nabla_{Y}Z\right)\\ & =g\left(Y,\left(\nabla_{X}J\right)Z\right)-g\left(X,\left(\nabla_{Y}J\right)Z\right)\\ & =g(Y,\tau (X)JZ)-g(X,\tau (Y)JZ)\\ & =-g(T(X,Y),JZ)\\ & =-\tilde{g}\left(T\left(X,Y\right),Z\right). \end{array}$$

□

**Proposition** **8.**  *Let (M,J,g) be an almost Norden manifold,* ∇ the Levi-Civita connection of g and $\left(\nabla ,\nabla^{*},\tilde{g}\right)$ a dualistic structure. If J is ∇*-recurrent, then for any X,Y,Z∈Γ(TM),*

*(i)*  $\nabla_{X}^{*}Y=\nabla_{X}Y+\tau \left(X\right)Y,$
*(ii)*  $\left(\nabla_{X}^{*}g\right)\left(Y,Z\right)=-2g\left(\tau \left(X\right)Y,Z\right),$
*(iii)*  $\left(\nabla_{X}^{*}g\right)\left(Y,Z\right)-\left(\nabla_{X}^{*}\tilde{g}\right)\left(Y,Z\right)=-\left(\nabla_{X}\tilde{g}\right)\left(Y,Z\right).$

**Proof.** 

(i) Let X,Y,Z∈Γ(TM). Then
  $$\begin{array}{c} X\tilde{g}\left(Y,Z\right)=\tilde{g}\left(\nabla_{X}Y,Z\right)+\tilde{g}\left(Y,\nabla_{X}^{*}Z\right), \end{array}$$
  $$\begin{array}{c} Xg\left(JY,Z\right)=g\left(J\left(\nabla_{X}Y\right),Z\right)+g\left(JY,\nabla_{X}^{*}Z\right), \end{array}$$
  $$\begin{array}{c} Xg\left(JY,Z\right)=-g\left(\left(\nabla_{X}J\right)Y,Z\right)+g\left(\nabla_{X}JY,Z\right)+g\left(JY,\nabla_{X}^{*}Z\right), \end{array}$$
  $$\begin{array}{c} g\left(JY,\nabla_{X}Z\right)=-g\left(\tau \left(X\right)JZ,Y\right)+g\left(JY,\nabla_{X}^{*}Z\right). \end{array}$$
  Thus
  $$\begin{array}{c} \nabla_{X}^{*}Z=\nabla_{X}Z+\tau \left(X\right)Z. \end{array}$$
(ii) By covariant differentiation of *g* with respect to $\nabla^{*}$, we have
  (13) $$\begin{array}{cc} \left(\nabla_{X}^{*}g\right)\left(Y,Z\right) & =\left(\nabla_{X}g\right)\left(Y,Z\right)-g\left(\tau \left(X\right)Y,Z\right)-g\left(Y,\tau \left(X\right)Z\right)\\ & =-2g(\tau (X)Y,Z). \end{array}$$
(iii) Covariant derivative of $\tilde{g}$ with respect to $\nabla^{*}$ is
  (14) $$\begin{array}{c} \left(\nabla_{X}^{*}\tilde{g}\right)\left(Y,Z\right)=\left(\nabla_{X}\tilde{g}\right)\left(Y,Z\right)-2g\left(\tau \left(X\right)Y,Z\right). \end{array}$$
  Subtracting (14) from (13),
  $$\begin{array}{c} \left(\nabla_{X}^{*}g\right)\left(Y,Z\right)-\left(\nabla_{X}^{*}\tilde{g}\right)\left(Y,Z\right)=-\left(\nabla_{X}\tilde{g}\right)\left(Y,Z\right). \end{array}$$

□

Denote by $\nabla_{X}^{J}Y=-J\nabla_{X}JY$, for any vector fields X,Y on *M*.

Before stating the next proposition, we recall the concept of projectively equivalent connections. Two linear connections ∇ and $\nabla^{P}$ on a differentiable manifold *M* are called projectively equivalent if there exists a 1-form τ such that

$$\begin{array}{c} \nabla_{X}^{P}Y=\nabla_{X}Y+\tau \left(X\right)Y+\tau \left(Y\right)X,\;\;\;\;\forall X,Y\in \Gamma \left(TM\right). \end{array}$$

**Proposition** **9.** *Let (M,J) be an almost complex manifold and* ∇ *and $\nabla^{P}$ projectively equivalent linear connections on M. If $\left(\nabla_{X}J\right)Y=\tau \left(X\right)JY$, then*

(15) $$\begin{array}{c} \nabla_{X}^{P}Y=\nabla_{X}^{J}Y-J\left(\nabla_{Y}J\right)X,\forall X,Y\in \Gamma \left(TM\right), \end{array}$$

(16) $$\begin{array}{c} R^{P}\left(X,Y\right)Z=R\left(X,Y\right)Z,\forall X,Y\in \Gamma \left(TM\right), \end{array}$$

*where $R^{P}$ is the curvature tensor of $\nabla^{P}$.*

**Proof.**  Based on $\left(\nabla_{X}J\right)Y=\tau \left(X\right)JY$, we can write

$$\begin{array}{cc} \nabla_{X}^{P}Y & =\nabla_{X}Y+\tau \left(X\right)Y+\tau \left(Y\right)X\\ & =\nabla_{X}Y-J\left(\nabla_{X}J\right)Y-J\left(\nabla_{Y}J\right)X\\ & =\nabla_{X}^{J}Y-J\left(\nabla_{Y}J\right)X. \end{array}$$

From (15) and using the skew-symmetry of the curvature, for any X,Y∈Γ(TM) we have

$$\begin{array}{c} \nabla_{X}^{P}Y=\nabla_{X}^{J}Y+\nabla_{Y}^{J}X-\nabla_{Y}X; \end{array}$$

thus we obtain

$$\begin{array}{c} R^{P}\left(X,Y\right)Z=R^{J}\left(X,Y\right)Z+R^{J}\left(Y,X\right)Z-R\left(Y,X\right)Z, \end{array}$$

$$\begin{array}{c} R^{P}\left(X,Y\right)Z=R\left(X,Y\right)Z. \end{array}$$

□

## 5. Example

We construct one complex statistical connection using a 1-form ρ given by $\rho \left(X\right)=\tilde{g}\left(X,\xi \right)$, where $\tilde{g}$ is the Norden metric and ξ is the dual vector field of ρ.

Since

$$\begin{array}{c} \rho \left(J\left(X\right)\right)=\left(\rho \circ J\right)X=\tilde{g}\left(JX,\xi \right)=g\left(JX,J\xi \right)=-g\left(X,\xi \right), \end{array}$$

and denoting by

$$\begin{array}{c} K(X,Y)=\rho (JX)\rho (JY)J(\xi ), \end{array}$$

we obtain the following.

$$\begin{array}{cc} \tilde{g}\left(K\left(X,Y\right),Z\right) & =\rho (JX)\rho (JY)g(J\xi ,JZ)\\ & =(-g(X,\xi ))(-g(Y,\xi ))(-g(Z,\xi ))\\ & =-g(Y,\rho (JX)\rho (JZ)\xi )\\ & =\tilde{g}\left(Y,\rho \left(JX\right)\rho \left(JZ\right)J\xi \right)\\ & =\tilde{g}\left(K\left(X,Z\right),Y\right). \end{array}$$

Then, the connection ∇, defined by

(17) $$\begin{array}{c} \nabla_{X}Y={\overset{\tilde{g}}{\nabla }}_{X}Y+\frac{1}{2}\rho \left(JX\right)\rho \left(JY\right)J\left(\xi \right), \end{array}$$

is a statistical connection, where $\overset{\tilde{g}}{\nabla }$ is the Levi-Civita connection of $\tilde{g}$. Now considering the statistical connection (17), we investigate some of its properties.

Let (M,J,g) be an almost Norden manifold with the Levi Civita connection ∇ such that ∇J=0. The triple (M,J,g) is called Kähler-Norden manifold.

**Proposition** **10.** *Let $\left(M,J,\tilde{g}\right)$ be a Kähler-Norden statistical manifold with the statistical connection (17). If ρ⊗J(ξ)=ρ∘J⊗ξ, then ∇J=0.*

**Proof.**  Since

$$\begin{array}{cc} (\nabla_{X}J)Y & =\nabla_{X}JY-J\left(\nabla_{X}Y\right)\\ & =\left({\overset{\tilde{g}}{\nabla }}_{X}J\right)Y-\frac{1}{2}\rho \left(JX\right)\rho \left(Y\right)J\left(\xi \right)+\frac{1}{2}\rho \left(JX\right)\rho \left(JY\right)\left(\xi \right), \end{array}$$

due to the assumption that *M* is Kähler-Norden, i.e., ${\overset{\tilde{g}}{\nabla }}_{X}Y=0$, and according to ρ⊗J(ξ)=ρ∘J⊗ξ, the proof is complete.   □

**Corollary** **3.** *Let $\left(M,J,\tilde{g}\right)$ be a Kähler-Norden statistical manifold with the statistical connection (17). If ρ⊗J(ξ)=ρ∘J⊗ξ, then*

K(JY,X)=K(JX,Y),∀X,Y∈Γ(TM).

Using (10) and (1), we reach the result.

**Proposition** **11.** *Let $\left(M,J,\tilde{g}\right)$ be a Kähler-Norden statistical manifold with the statistical connection (17). Then, for any X,Y∈Γ(TM),*

$$\begin{array}{c} K\left(JY,X\right)=K\left(JX,Y\right)\Leftrightarrow \left(\nabla_{X}J\right)Y=\left(\nabla_{Y}J\right)X. \end{array}$$

**Proof.**  From

$$\begin{array}{c} \left(\nabla_{X}J\right)Y=\left({\overset{\tilde{g}}{\nabla }}_{X}J\right)Y\frac{1}{2}-\rho \left(JX\right)\rho \left(Y\right)J\left(\xi \right)+\frac{1}{2}\rho \left(JX\right)\rho \left(JY\right)\left(\xi \right), \end{array}$$

and

$$\begin{array}{c} \left(\nabla_{Y}J\right)X=\left({\overset{\tilde{g}}{\nabla }}_{Y}J\right)X-\frac{1}{2}\rho \left(X\right)\rho \left(JY\right)J\left(\xi \right)+\frac{1}{2}\rho \left(JY\right)\rho \left(JX\right)\left(\xi \right), \end{array}$$

we have

$$\begin{array}{c} K\left(JY,X\right)-K\left(JX,Y\right)=\left(\nabla_{X}J\right)Y-\left(\nabla_{Y}J\right)X. \end{array}$$

□

**Corollary** **4.**  *Let $\left(M,J,\tilde{g}\right)$ be a Kähler-Norden statistical manifold with the statistical connection (17). Then the following conditions are equivalent.*

*(i)* 
K(JY,X)=K(JX,Y),∀X,Y∈Γ(TM),

*(ii)*  *$\left(\nabla_{X}J\right)Y=\left(\nabla_{Y}J\right)X,\forall X,Y\in \Gamma \left(TM\right)$,*
*(iii)* 
$\left(\nabla_{X}^{*}J\right)Y=\left(\nabla_{Y}^{*}J\right)X,\forall X,Y\in \Gamma \left(TM\right).$

Proposition 11 and Corollary 4 imply the following.

**Proposition** **12.**  *Let $\left(M,J,\tilde{g}\right)$ be an almost Norden statistical manifold with the statistical connection (17). Then any two of the following conditions imply the third one.*

*(i)* 
K(JY,X)=K(JX,Y),∀X,Y∈Γ(TM),

*(ii)* 
$\left(\nabla_{X}J\right)Y=\left(\nabla_{Y}J\right)X,\forall X,Y\in \Gamma \left(TM\right),$

*(iii)* 
$\left({\overset{\tilde{g}}{\nabla }}_{X}J\right)Y=\left({\overset{\tilde{g}}{\nabla }}_{Y}J\right)X,\forall X,Y\in \Gamma \left(TM\right).$
